# Panel Fees: A Question of Ethics

**Published:** 1924-03

**Authors:** 


### PANEL FEES: A QUESTION OF ETHICS.

The Panel doctors may, perhaps, be a little extravagant in expression in speaking of the finding of the Court of Enquiry, in favour of a 9s. capitation fee, as a victory. But they probably have in mind not so much the 8s. 6d. which was offered by the Minister as the smaller sums ranging from 7s. 3d. to 8s. which some of the approved societies' spokesmen seemed to consider adequate. We may, at least, hope that the question is removed for some time from the region of controversy; this would be good both for the service and for the doctors themselves.

### When may Private Treatment be Given?

Before decently interring this subject of the doctors' fees—although with a fear that there may be a later exhumation—it may be convenient to remind doctors and insured persons alike of the comprehensive character of the Panel doctor's contract. The doctors collectively have assumed a responsibility for the treatment of all insured persons. Doctors are, however, very human, and a question which is constantly being asked by individual doctors is this: Am I entitled to treat certain insured persons as private patients? The reply in the first instance to this question might very well be another question: What sort of treatment do you propose to give to these insured persons as private patients which you would otherwise refuse to them?

### What the Regulations Say.

If a doctor proves that he possesses special qualifications, he may quite properly charge an insured person for specialist services. But usually the enquiry relates to treatment which is admittedly within the general range of the Panel doctor's duties. Under his terms of service, the doctor "is not permitted to demand or accept any fee or other remuneration in respect of treatment which he is required to give." He can charge a fee by way of returnable deposit when he is in doubt whether the applicant for treatment is an insured person, but we are dealing now with the case where the insured person specifically asks for treatment as a private patient.

### Where the Doctor's Obligations Arise.

The whole of a doctor's obligations under his Insurance contract towards any individual patient arise at the moment when that patient "in applying for treatment represents that he is an insured person." If the doctor accepts, or has accepted, that insured person on his list, there can be no question that he is debarred from accepting any fee from him. If, however, the insured person is on the list of another doctor, the first doctor is under no obligation to treat him, except in an emergency when his own doctor is not available, and if he does accept him as a private patient he is entitled to charge a fee for doing so. But the obvious course would be that he should be transferred to that doctor's list; all that this involves is the signing of his medical card by the new doctor. Except in those rare instances where the patient has come for a second opinion and bonâ fide wants to remain on the list of his old doctor, and normally to continue to receive treatment from him, the case for treating another doctor's patients as private patients is really not distinguishable from that which we will now consider where the patient is not on any doctor's list.

### The Undeclared Patient.

In the case even of a new patient a doctor should rarely have trouble in determining whether the applicant is an insured person or not. Every manual worker, and every other employed person not earning more than £250 a year, is insured, and every insured person as such is entitled to medical benefit. It is a mistaken, and in some cases a mean policy, for a doctor to assume in every case that an applicant is a private patient, unless he expressly states that he is an insured person. The terms of service now contain an important provision that, in all such cases, the patient may within one month apply for repayment of the doctor's fee or the withdrawal of his account. This will put the matter right where there has been a genuine misunderstanding.

### The Patient Who Insists.

But where the applicant deliberately elects to forgo the benefit to which he is entitled under the Act, and insists upon being treated as a private patient, what is the doctor to do? It may fairly be argued that although the applicant makes it known to the doctor, whether reluctantly or not, that he is an insured person, this is not a representation as part of his application for treatment that he is an insured person. This is, perhaps, a little thin as a legal argument, but it would not appear that the regulations contemplate that a man should be compelled to receive treatment as an insured person from the doctor of his choice, or in the alternative to go elsewhere for treatment. It may therefore be said that by charging such a person a fee, the Panel doctor is not committing a breach of the terms of service. That is about all that can be said in favour of such a course.

### The Good Name of the Service.

It is clear that a doctor who gives treatment for a fee—except in the case of specialist services—on the ground that the insurance service is an inferior service would not be allowed to remain on the panel. If we free our minds from cant, it is only on such a ground that an insured person would press for treatment as a private patient. Such an application places the doctor in a very unfair position, but for his own good name and that of the service which he has entered, his only safe rule of conduct is to refuse to be a party to any arrangement of the kind, even if this involves the loss of a few fees. It is, perhaps, as a question of ethics that most doctors will rightly solve this problem for themselves.

---

**Hospital Grants.**

The Board of Delegates of the Metropolitan Hospital Saturday Fund has made grants amounting to £71,532 to 225 London institutions.

